# The author’s response

**DOI:** 10.1186/s40560-021-00537-9

**Published:** 2021-03-16

**Authors:** Yohei Hirano, Shunsuke Madokoro, Yutaka Kondo, Ken Okamoto, Hiroshi Tanaka

**Affiliations:** Department of Emergency and Critical Care Medicine, Juntendo University Urayasu Hospital, 2-1-1 Tomioka, Urayasu, Chiba 279-0021 Japan

We thank you for your interest in our study which performed systematic review and meta-analysis of randomized controlled trials (RCTs) on the effect of corticosteroid treatment initiated in an early stage of acute respiratory distress syndrome (ARDS) [1]. We are willing to provide our response to address your concerns.

As we described in Additional file 2, the report by Annanne et al., one of the studies included in our meta-analysis, was a post hoc analysis of the RCT [2]. The original RCT targeted on patients with septic shock [3]. Although we included this report in our meta-analysis, it was interpreted with special caution taking its specific population of ARDS into consideration. We excluded the RCT from Liu et al. [4]. As shown in Figure 1, 2 in our article [1], RCTs that were not published in English have been excluded from the screening process of full-text articles in our meta-analysis. Moreover, the inclusion criteria of the RCT by Liu et al. were ARDS patients combined with critical illness-related corticosteroid insufficiency (CIRCI), which obviously exhibits a specific population to assess the effect of corticosteroids. The RCT by Rezk et al. was also excluded due to the different setting of its outcome; while our primary outcome was 28- or 30-day mortality, they only showed 7- or 14-day mortality in their report.

For the primary outcome in our meta-analysis, the optimal information size (OIS) criteria were met when we set the relative risk reduction at 30%. Thus, we did not consider the imprecision to be “serious.” However, as the recommended setting of relative risk reduction ranges between 20 and 30%, it is possible that someone who set it at 20% sees higher imprecision risk in it.

We realize that there are still several limitations in our study. The possibility of publication bias was described in the discussion (Limitations of the study) because the reliability of Egger’s linear regression test is weak due to the limited number of RCTs covered in it. Although we carefully downgraded the certainty of evidence because it should not be made in the mathematical manner, it is understandable if someone would assess it even lower taking these concerns more seriously.

Nevertheless, we believe that the current meta-analysis would provide valuable information for clinical practitioners to help them decide on early corticosteroid treatment on ARDS. Attention should be paid to the initiated timing of corticosteroids. Although it is true that this treatment still remains controversial and yet to be improved, the current meta-analysis, as well as accumulating evidence on the efficacy of corticosteroid treatment in COVID-19, would accelerate the future invention of better-designed trials that might clear uncertainty of the evidence on corticosteroid therapy in ARDS.
